# S134. THE INCIDENCE OF PSYCHOSIS IN OLDER PEOPLE: A SWEDISH POPULATION-BASED COHORT STUDY

**DOI:** 10.1093/schbul/sby018.921

**Published:** 2018-04-01

**Authors:** Jean Stafford, Robert Howard, Christina Dalman, James Kirkbride

**Affiliations:** 1 University College London; 2 Karolinska Institutet

## Abstract

**Background:**

People aged 65 and above have consistently been omitted from research on the epidemiology of psychotic disorders. Correspondingly, little is known about the incidence of very late-onset schizophrenia-like psychosis (VLOSLP). We aimed to characterise the incidence of VLOSLP in a Swedish population cohort, including how incidence varied by age, sex, migration, deprivation, traumatic life events, and social isolation.

**Methods:**

We conducted a Swedish population-based cohort study to examine the incidence of VLOSLP by potential environmental risk factors. The cohort, born in 1920–1949 and living in Sweden, were followed up from age 60 until the end of follow-up (30th December 2011), emigration, death, or diagnosis with a non-affective psychotic disorder. We used Cox regression to obtain hazard ratios and 95% confidence intervals for VLOSLP by age, sex, migration, disposable income at age 60, and the experience of the death of a partner or child, adjusting for potential confounders.

**Results:**

In a cohort of 2,955,796 people, we identified 14,825 cases with VLOSLP, with an overall incidence rate of 38.1 (95% CI: 37.5 – 38.7) per 100,000 person-years at-risk. Rates were higher amongst migrants from North America (HR=1.4, 95% CI=1.0–1.9), Europe (HR=1.5, 95% CI=1.4–1.6), Russian-Baltic regions (HR=1.7, 95% CI=1.4–2) and Africa (HR=2.0, 95% CI=1.4–2.7) compared to Swedish-born, with a lower rate in migrants from the Middle East (HR=0.7, 95% CI=0.5–1.0). Rates were higher in those with the lowest income (HR=3.1, 95% CI=2.9–3.3), who experienced the death of a partner (HR=1.1, 95% CI=1.0–1.2), death of a child in infancy (HR=1.2, 95% CI=1.0–1.5), and those without a partner (HR=1.8, 95% CI=1.8–1.9) or children (HR=2.6, 95% CI=2.5–2.7).

**Discussion:**

In this large, national cohort study we identified several potential risk factors for developing psychosis later in life, including migration, deprivation, social isolation and traumatic life events. This may have important implications for our understanding of the aetiology of VLOSLP and could help to inform public mental health and service planning.

